# Western Diet-Induced Impairment of Left Atrium Cardiomyocyte Contractility in Female Wistar Rats Is Associated with Slowdown in the Cross-Bridge Cycle and Dephosphorylation of cMyBP-C

**DOI:** 10.3390/ijms27031508

**Published:** 2026-02-03

**Authors:** Elena Mukhlynina, Xenia Butova, Tatiana Myachina, Raisa Simonova, Yulia Antonets, Anna Leiberova, Anastasia Kochurova, Evgeniya Gusarova, Tatiana Chumarnaya, Galina Kopylova, Daniil Shchepkin

**Affiliations:** 1 Institute of Immunology and Physiology, Russian Academy of Sciences, Pervomayskaya Str. 106, 620078 Yekaterinburg, Russia; myachina.93@mail.ru (T.M.); raisa.simonova@mail.ru (R.S.); uliaantonec@gmail.com (Y.A.); aleyberova@gmail.com (A.L.); kochurova.a.m@mail.ru (A.K.); evgeniyagusarova89@yandex.ru (E.G.); chumarnaya@gmail.com (T.C.); g_rodionova@mail.ru (G.K.); 2 Institute of Biomedical Problems RAS, Khoroshevskoye sh., 76A, 123007 Moscow, Russia; x.butova@gmail.com; 3 Regional Perinatal Center, Vorovskogo Str. 70, 454052 Chelyabinsk, Russia

**Keywords:** Western diet, obesity, left and right atria, echocardiography, single cardiomyocyte mechanics, calcium transient, actin–myosin interaction, contractile protein phosphorylation

## Abstract

Obesity is a major risk factor for heart failure and atrial fibrillation. This study investigated the effects of diet-induced obesity on the molecular and cellular mechanisms of cardiomyocyte contractility in the left and right atria (LA and RA). Female Wistar rats were fed a Western diet (WD) for 18 weeks. Sarcomere dynamics and calcium transients were measured in unloaded cardiomyocytes. Actin–myosin interactions and contractile protein phosphorylation were assessed via an in vitro motility assay and phosphoprotein-specific gel electrophoresis. WD-fed rats developed obesity, hypertension, and metabolic alterations in the absence of echocardiographic or histological evidence of cardiac remodeling or systolic dysfunction. In LA cardiomyocytes, contractile dysfunction was indicated by increased calcium transient amplitude coupled with reduced shortening amplitude and relengthening velocity. This functional impairment correlated with a slowed myosin cross-bridge cycle and dephosphorylation of cMyBP-C. In contrast, RA cardiomyocytes displayed only molecular changes in response to obesity, including altered phosphorylation of most sarcomeric proteins and a decelerated cross-bridge cycle, but showed no evident contractile dysfunction. Thus, an 18-week WD reflects the early stages of contractile impairment, where functional deficits are specific to the LA, while RA alterations are confined to the molecular level.

## 1. Introduction

Nowadays, according to some estimates, the global prevalence of metabolic syndrome (MetS) reaches 31.4% and correlates with a country’s income level [1]. In addition to genetics, obesity is driven by various lifestyle and dietary factors. The prevalence of overweight is negatively impacted by a “Western” diet (WD), which is high in saturated fats and simple sugars [2,3]. Obesity and MetS are recognized as leading risk factors for cardiovascular diseases (CVDs) [4], including myocardial fibrosis and atrial fibrillation [5] in both men and women [6]. Furthermore, obesity and diabetes are associated with heart failure with preserved ejection fraction (HFpEF), a condition characterized by diastolic dysfunction [7].

In obesity, a variety of structural and functional alterations in the heart have been observed. Maugeri et al. [8] found that WD exposure increases the risk of concentric left ventricular (LV) hypertrophy in humans. In mice, WD feeding induces diastolic and systolic dysfunction, as well as LV remodeling [9], cardiomyocyte hypertrophy [10], a significant increase in collagen I and collagen IV gene expression [11], right ventricular (RV) hypertrophy, and increased RV systolic pressure [12]. In rats, the WD also promotes increased systolic blood pressure (SBP) and cardiac remodeling, including enlargement of the left atrium and LV diastolic and systolic diameter, as well as deterioration of cardiac function, as visualized by echocardiographic analysis [13]. Additionally, Stepanyan et al. [14] highlighted variations in the cardiac response to WD depending on age and sex. Male mice exhibited no detectable structural changes in the LV but developed heart failure with reduced ejection fraction (HFrEF), i.e., systolic dysfunction, whereas females demonstrated more pronounced structural alterations and echocardiographic signs of diastolic stiffness [14].

Notably, research attention has primarily focused on the structural and functional characteristics of the ventricles—especially the left ventricle (LV)—during exposure to a WD. It has been found that in rats, high-fat diet (HFD)-induced obesity leads to increased systolic sarcomere shortening and a higher amplitude of cytosolic Ca^2+^ transients in ventricular cardiomyocytes [15]. Studies in mice fed a high-fat/high-sugar diet (HFD/HSD) demonstrated that under loaded conditions, the maximal sarcomere lengthening rate of cardiomyocytes decreases; upon stretching, the Ca^2+^ transient decay time course is prolonged, and cardiomyocyte stiffness increases, which is associated with diastolic dysfunction [16]. Aslam et al. [17] found reduced sarcomere contractility in cardiomyocytes from RV biopsies of obese patients with HFpEF. The authors showed that while the active tension of cardiomyocytes decreases, both passive tension and the Ca^2+^ sensitivity of tension increase.

The contractile function of atrial cardiomyocytes and the role of sarcomeric proteins have remained largely unexplored. Only a few studies have been devoted to atrial contractile function in MetS. In the work of Huettemeister et al. [18], an increase in cell size and T-tubule density, along with an increased number of mitochondria, was demonstrated using cardiomyocytes isolated from patients with obesity or diabetes mellitus. Echocardiographic studies of obese patients have revealed that a high body mass index is associated with early subclinical changes in right atrial trabecular function, characterized by delayed relaxation and reduced adrenergic lusitropy [19]. In the ZSF-1 rat model of MetS, the development of HFpEF was shown to be associated with a significantly increased left atrial area and impaired atrial function in vivo, as well as changes in cardiomyocyte calcium signaling. However, the mechanical properties of atrial cardiomyocytes were not assessed [20].

Significant sex differences in cardiovascular risk and outcomes are well-established [21]. The mechanisms driving CVDs are also presumed to have sex-specific characteristics [22]. However, female animals are underrepresented in preclinical research. Recognizing sex-related differences in the development of MetS-associated early contractile disorders is essential for understanding the pathogenesis of CVDs, as well as for developing effective, tailored prevention and treatment strategies.

Consequently, there are significant gaps in the knowledge regarding sex-specific early-stage changes in the mechanical function of left and right atrial cardiomyocytes driven by obesity and metabolic disturbances. The aim of this study was to investigate the impact of obesity on the molecular and cellular mechanisms of atrial myocardial contraction in female rats. We used a dietary WD model and analyzed sarcomere shortening–relengthening dynamics and cytosolic calcium ([Ca^2+^]_i_) transients in contracting cardiomyocytes isolated from the left (LA) and right (RA) atria. To explore potential mechanisms underlying the effects of MetS on the contractile properties of LA and RA cardiomyocytes, we assessed the characteristics of actin–myosin interaction using an in vitro motility assay and evaluated the phosphorylation levels of key sarcomeric proteins.

## 2. Results

### 2.1. Obesity as a Result of WD

**Figure 1:**
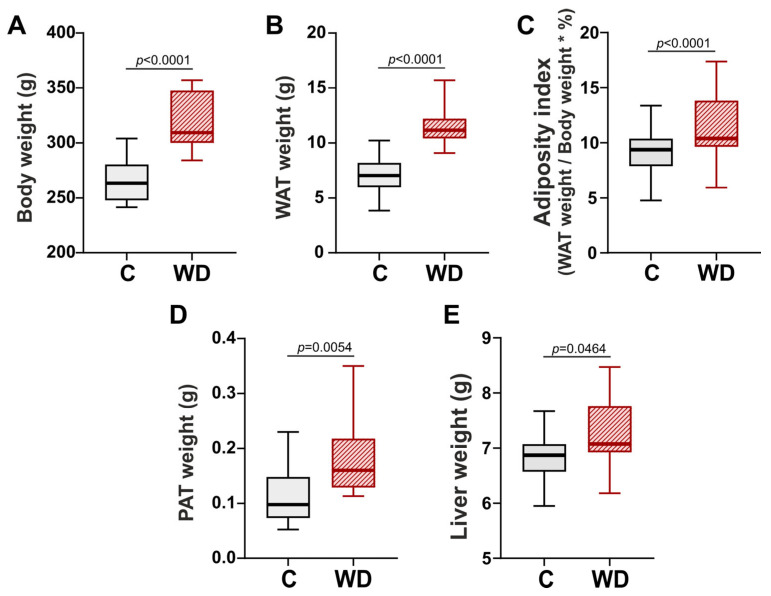
Western diet (WD) promoted obesity and liver weight gain in female rats. (**A**) Body weight. (**B**) Total white adipose tissue (WAT). (**C**) Adiposity index (WAT weight/body weight ∗ 100%). (**D**) Pericardial adipose tissue (PAT) weight. (**E**) Liver weight. Data are presented in box and whisker plots: the boxes are drawn from Q1 to Q3, bold lines show medians, and whiskers provide the 100% ranges of the values. N = 12 rats per group. Mann–Whitney U-test, *p* < 0.05.

Obesity is characterized by an excessive accumulation of fat within the body. Therefore, the animal’s body weights, abdominal white adipose tissue (WAT), pericardiac adipose tissue (PAT), and livers were measured. After 18 weeks WD-fed female rats were 17.5% heavier than those in the control group (Figure 1A). Additionally, a significant increase in total WAT mass (by 89.6%), adiposity index (by 58.7%), PAT (by 514.4%) and liver weight (by 3.0%) was observed in the WD group compared to the control (Figure 1B–E).

### 2.2. Violation of Glucose Homeostasis

**Figure 2:**
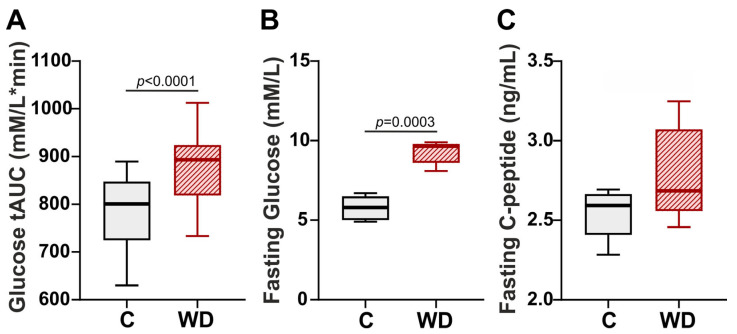
Effects of Western diet (WD) on glucose homeostasis in female rats. (**A**) Total area under the curve (tAUC) for glucose concentration in oral glucose tolerance test after 16 weeks of feeding. (**B**) Plasma fasting glucose. (**C**) Plasma fasting C-peptide. Data are presented in box and whisker plots: the boxes are drawn from Q1 to Q3, bold lines show medians, and whiskers provide the 100% ranges of the values. N = 12 rats per group. Mann–Whitney U-test, *p* < 0.05.

As shown in Figure 2, after 16 weeks of WD rats exhibited a reduced ability to utilize glucose compared to the control group. In the oral glucose tolerance test (OGTT), rats fed a WD experienced a more pronounced increase in blood glucose levels, followed by a slower decline compared to the control group. The total area under the curve (AUC) in the OGTT for the WD-exposed animals was 11.5% greater than that for the control group (Figure 2A). In addition, a moderate increase in fasting plasma glucose levels (by 66.4%) was observed in rats after 18 weeks of the WD (Figure 2B). However, we found no impact of WD on plasma C-peptide levels (Figure 2C).

### 2.3. Alterations in the Lipid Profile

**Figure 3:**
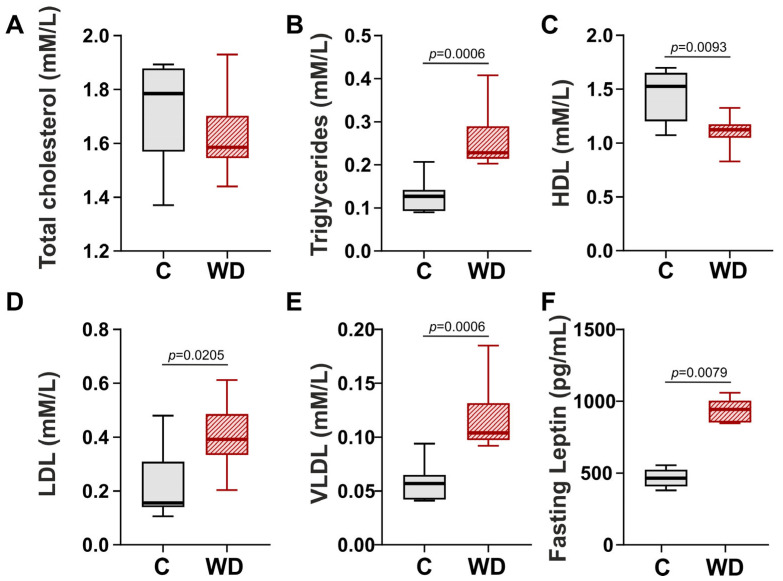
Effects of Western diet (WD) on lipid homeostasis in female rats. (**A**) Plasma fasting total cholesterol. (**B**) Plasma fasting triglycerides. (**C**) Plasma fasting high-density lipoprotein (HDL) cholesterol. (**D**) Plasma fasting low-density lipoprotein (LDL) cholesterol. (**E**) Plasma fasting very low-density lipoprotein (VLDL) cholesterol. (**F**) Plasma fasting leptin. Data are presented in box and whisker plots: the boxes are drawn from Q1 to Q3, bold lines show medians, and whiskers provide the 100% ranges of the values. N = 8 rats per group. Mann–Whitney U-test, *p* < 0.05.

Obesity often correlates with dyslipidemia. Consequently, we assessed lipid metabolism in WD-fed rats. Alterations in the lipid profile were evident in the WD group (Figure 3). WD did not affect plasma total cholesterol levels (Figure 3A). However, plasma fasting triglycerides increased by 79.9% and high-density lipoprotein (HDL) decrease by 26.4% compared to the control group (Figure 3B,C). Low-density lipoproteins (LDLs) and very-low-density lipoproteins (VLDLs) estimated by the Friedewald formula were significantly elevated (by 151.0% and 82.5%) compared to those in the control group (Figure 3D,E). In addition, WD-fed rats had significantly higher fasting leptin levels (by 103.1%) than the control group (Figure 3F).

### 2.4. Hemodynamic and Echocardiographic Parameters

**Figure 4:**
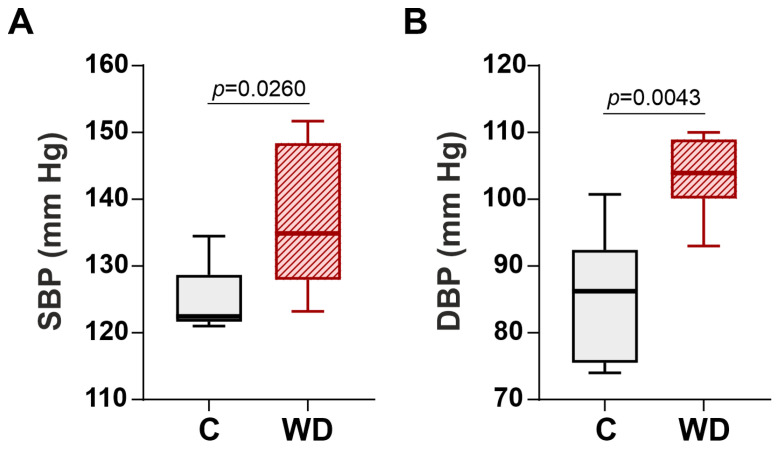
Effects of Western diet (WD) on systolic (SBP) and diastolic blood pressure (DBP) in female rats. (**A**) SBP. (**B**) DBP. Data are presented in box and whisker plots: the boxes are drawn from Q1 to Q3, bold lines show medians, and whiskers provide the 100% ranges of the values. N = 6 rats per group. Mann–Whitney U-test, *p* < 0.05.

In the WD group, there was a significant elevation of the SBP (Figure 4A) and diastolic blood pressure (DBP) (Figure 4B) by 10.1% and 20.5% respectively compared to the control group. These findings indicate that WD was able to trigger hypertension in the female Wistar rats.

However, we did not find any significant differences between the WD and control groups in the characteristics of the standard echocardiographic protocol (Table 1).

### 2.5. Histological Characteristics of the LA and RA Myocardium and Atrial Cardiomyocyte Morphometry

**Figure 5:**
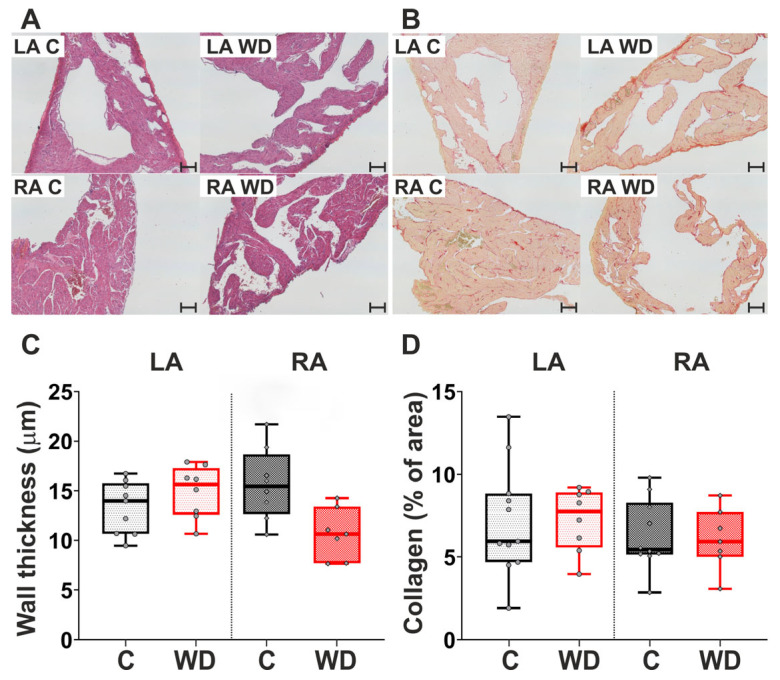
Effect of Western diet (WD) on the atrial histology in female rats. (**A**) Representative microphotographs of the atrial frontal sections stained by hematoxylin and eosin (H&E) for assessment of wall thickness. (**B**) Representative microphotographs of the atrial frontal sections stained by picrosirius red for assessment of collagen content. Objective magnification 10×. Control segment—100 µm. (**C**) Atrial wall thickness. (**D**) Atrial collagen content. LA—left atrium; RA—right atrium. Data are presented in box and whisker plots: the boxes are drawn from Q1 to Q3, bold lines show medians, and whiskers provide the 100% ranges of the values. Each dot represents value an individual animal (N = 9 rats per group). Mann–Whitney U-test, *p* < 0.05.

Representative areas of atrial myocardium from the control and WD groups, stained with hematoxylin and eosin (H&E) and picrosirius red, are presented in Figure 5A,B. Histological analysis revealed no significant difference in LA and RA wall thickness between rats fed the WD and the control group (Figure 5A,C). The collagen content of both the LA and RA remained largely unchanged in the WD group (Figure 5B,D).

**Figure 6:**
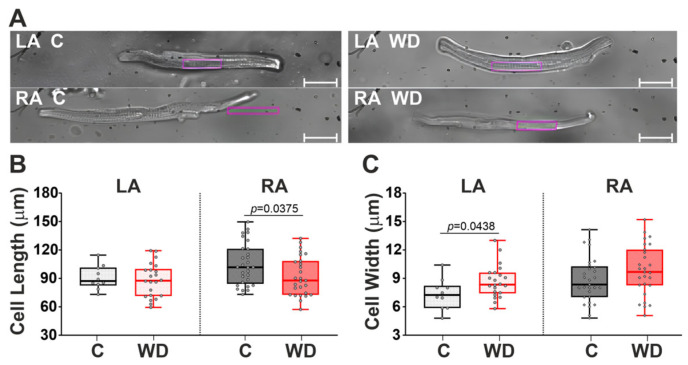
Effects of Western diet (WD) on morphometry of single cardiomyocytes from the LA and RA. (**A**) Representative images of single isolated cardiomyocytes showing WD-induced changes in cell length and width. (**B**) Cardiomyocyte length. (**C**) Cardiomyocyte width. Objective magnification 40×. Control segment—10 µm. LA—cardiomyocytes of the left atrium; RA—cardiomyocytes of the right atrium. Data are presented in box and whisker plots: the boxes are drawn from Q1 to Q3, bold lines show medians, and whiskers provide the 100% ranges of the values. Each dot represents value of individual cardiomyocyte (N = 6 rats per group). One-way ANOVA followed by Sidak’s post hoc test, *p* < 0.05.

Then, we estimated the effect of the WD on the morphometry of single isolated cardiomyocytes. Representative photos of isolated LA and RA cardiomyocytes are shown in Figure 6A. In the WD group, we observed a 14.1% decrease in cardiomyocyte length in the RA and a 15.1% increase in cardiomyocyte width in the LA, indicating the development of atrial hypertrophy (Figure 6B,C).

Thus, the 18-week WD in female rats has a significant effect on single cardiomyocyte morphology.

### 2.6. Parameters of Sarcomere Length Dynamics in Single Atrial Cardiomyocytes

**Figure 7:**
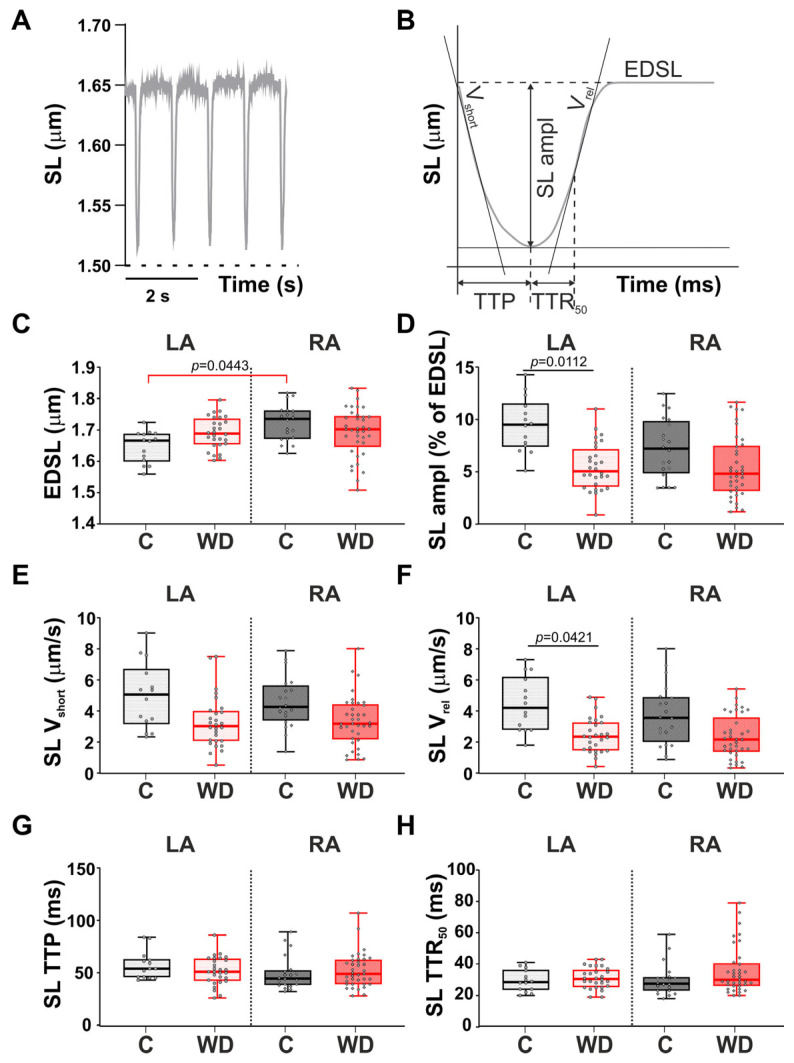
Effects of Western diet (WD) on sarcomere length (SL) dynamics in single mechanically non-loaded atrial cardiomyocytes. (**A**) Representative recordings of the time-dependent changes in SL dynamics in single contracting atrial cardiomyocytes. (**B**) Analyzed parameters derived from the average SL profile. (**C**) End-diastolic SL (EDSL). (**D**) Fraction SL shortening amplitude (in % of EDSL, SL ampl). (**E**) Maximum velocity of SL shortening (SL v_short_). (**F**) Maximum velocity of SL relengthening (SL v_rel_). (**G**) Time to peak sarcomere shortening (SL TTP). (**H**) Time to 50% sarcomere relengthening (SL TTR_50_). LA—cardiomyocytes of the left atrium; RA—cardiomyocytes of the right atrium. Data are presented in box and whisker plots: the boxes are drawn from Q1 to Q3, bold lines show medians, and whiskers provide the 100% ranges of the values. Each dot represents the value of an individual cardiomyocyte (N = 6 rats per group). Nested ANOVA followed by Sidak’s post hoc test, *p* < 0.05.

The studied parameters of sarcomere length (SL) dynamics in single mechanically non-loaded atrial cardiomyocytes are presented in Figure 7A,B. WD influenced SL dynamics exclusively in LA cardiomyocytes. In the LA of the WD-fed rats, the absolute (Figure S1 in the Supplementary Materials) and fractional SL shortening amplitude was reduced by 43.3% and 46.85%, respectively, and the maximum SL relengthening velocity declined by 44.9% (Figure 7D,F). The end-diastolic sarcomere length (EDSL), the LA time-course parameters of the SL shortening (time to peak sarcomere shortening, SL TTP, and time to 50% sarcomere relengthening, SL TTR_50_) did not change in the WD group relative to the control.

Thus, the sarcomere dynamics of the LA are significantly more sensitive to WD compared to the RA, which manifests in SL contractile dysfunction and impaired sarcomere relaxation.

### 2.7. Parameters of Cytosolic [Ca^2+^]_i_ in WD

**Figure 8:**
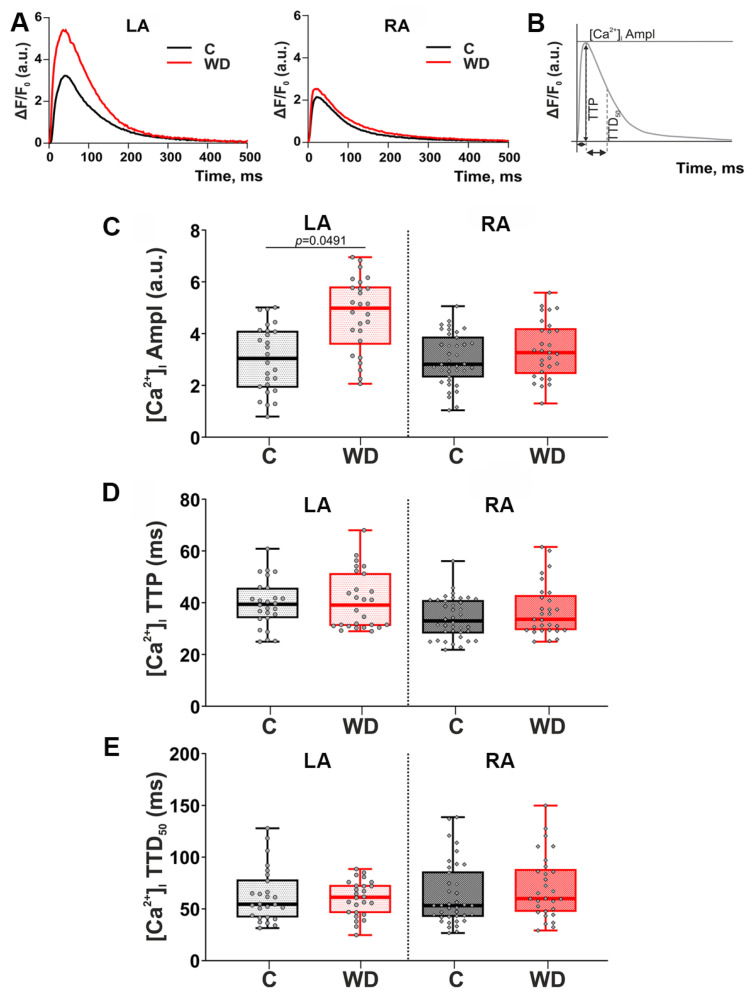
Effects of Western diet (WD) on cytosolic [Ca^2+^]_i_ transients in single mechanically non-loaded atrial cardiomyocytes. (**A**) Representative recordings of [Ca^2+^]_i_ transients in LA and RA cardiomyocytes. (**B**) Analyzed parameters derived from the [Ca^2+^]_i_ fluorescent signal. (**C**) An amplitude of [Ca^2+^]_i_ transients ([Ca^2+^]_i_ Ampl). (**D**) Time to peak [Ca^2+^]_i_ transients ([Ca^2+^]_i_ TTP). (**E**) Time to 50% [Ca^2+^]_i_ decay ([Ca^2+^]_i_ TTD_50_). LA—cardiomyocytes of the left atrium; RA—cardiomyocytes of the right atrium. Data are presented in box and whisker plots: the boxes are drawn from Q1 to Q3, bold lines show medians, and whiskers provide the 100% ranges of the values. Each dot represents the value of an individual cardiomyocyte (N = 5 rats per group). Nested ANOVA followed by Sidak’s post hoc test, *p* < 0.05.

The studied parameters of cytosolic [Ca^2+^]_i_ are presented in Figure 8A,B. In the LA of the WD group, cytosolic [Ca^2+^]_i_ transient amplitude was increased by 63.8% (Figure 8C). Time-course parameters of cytosolic [Ca^2+^]_i_ transients (time to peak [Ca^2+^]_i_ transients, [Ca^2+^]_i_ TTP, and time to 50% [Ca^2+^]_i_ decay, [Ca^2+^]_i_ TTD_50_) in the WD group did not change (Figure 8D,E).

Thus, WD affects only the cytosolic [Ca^2+^]_i_ transient amplitude in LA cardiomyocytes. This set of results is consistent with the SL dynamics data, where the WD effect is seen only in LA cardiomyocytes.

### 2.8. Actin–Myosin Interaction in the Atria

**Figure 9:**
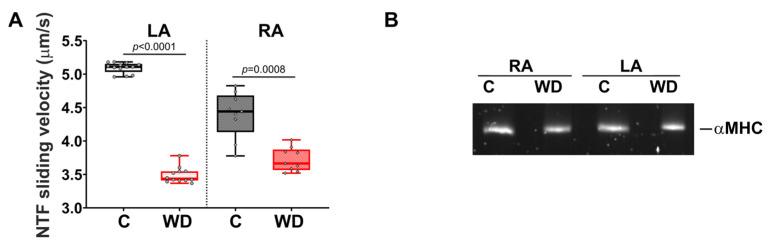
Effects of Western diet (WD) on actin–myosin interaction in the atria. (**A**) Sliding velocity of native thin filaments (NTFs) over myosin in the in vitro motility assay. (**B**) Isoform composition of myosin heavy chains (MHCs). Gels were stained by SYPRO Ruby. LA—left atrium; RA—right atrium. Data are presented in box and whisker plots: the boxes are drawn from Q1 to Q3, bold lines show medians, and whiskers provide the 100% ranges of the values. Each dot represents the average value for one animal (N > 5 rats per group). One-way ANOVA followed by Sidak’s post hoc test, *p* < 0.05.

In the WD group, the sliding velocity of native thin filaments (NTFs) on myosin in the in vitro motility assay decreased by 32.7% for the LA and by 17.5% for the RA compared with the control group (Figure 9A). Note that in all the studied groups, heavy-chain isoforms of myosin (MHCs) from the RA and LA were represented by αMHCs (Figure 9B).

### 2.9. Phosphorylation of Thin and Thick Filament Proteins

**Figure 10:**
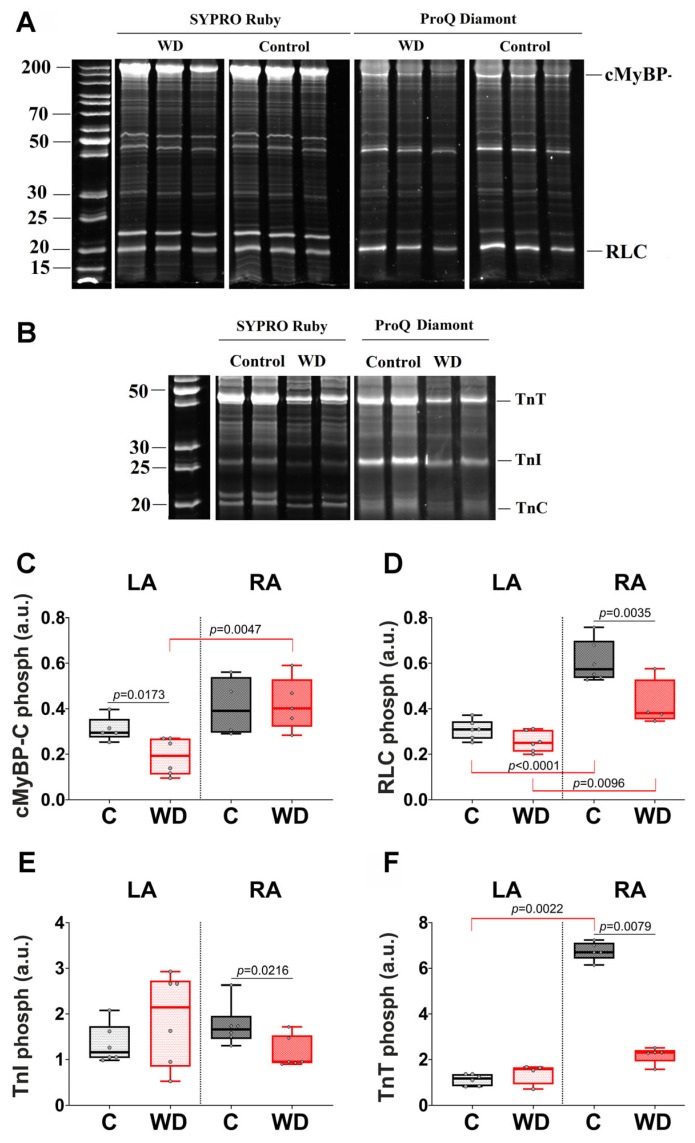
Effects of Western diet (WD) on phosphorylation levels of contractile sarcomeric proteins. (**A**) Examples of replicate gels for determining the degree of cMyBP-C and RLC phosphorylation in the LA. (**B**) Examples of replicate gels for determining the degree of TnT and TnI phosphorylation in the RA. PageRuler™ Unstained Protein Ladder (Thermo Fisher Scientific, Inc., Waltham, MA, USA) was used as a molecular weight marker for proteins. (**C**) cMyBP-C phosphorylation. (**D**) RLC phosphorylation. (**E**) TnI phosphorylation. (**F**) TnT phosphorylation. cMyBP-C—cardiac myosin-binding protein-C; RLC—myosin regulatory light chain; TnT—troponin T; TnI—troponin I; LA—left atrium; RA—right atrium. Data are presented in box and whisker plots: the boxes are drawn from Q1 to Q3, bold lines show medians, and whiskers provide the 100% ranges of the values. Each dot represents the average value for one animal (N > 5 rats per group). One-way ANOVA followed by Sidak’s post hoc test, *p* < 0.05.

The WD had different effects on sarcomere protein phosphorylation in the LA and RA (Figure 10). In the LA, WD led to a 34.6% decrease in cardiac myosin-binding protein C (cMyBP-C) phosphorylation compared to the control group (Figure 10C). Meanwhile, phosphorylation of myosin regulatory light chains (RLCs), troponin T (TnT), and troponin I (TnI) in the LA from the WD group remained at the control level. In the RA, WD resulted in a decrease in RLC, TnI and TnT phosphorylation by 33.5%, 42.6% and 65.6%, respectively (Figure 10D–F).

An additional effect of WD on the phosphorylation profile of the sarcomeric proteins should be noticed. In control rats, RA had higher levels of RLC and TnT phosphorylation than LA (85.57% and 470%, respectively), whereas cMyBP-C phosphorylation did not differ between the atria. In the WD group, we observed a 108% higher level of cMyBP-C phosphorylation in the RA and elimination of the interatrial differences for TnT phosphorylation.

Thus, the WD leads to more pronounced disturbances in the phosphorylation of sarcomere proteins in the RA, which is accompanied by a change in the phosphorylation profile between the LA and the RA.

## 3. Discussion

Our study aimed to investigate the effects of the WD on the contractile function of atrial cardiomyocytes in female rats, providing valuable insights into the early stages of heart failure pathogenesis, particularly atrial fibrillation, which is more prevalent in individuals with obesity and associated metabolic disorders. A dietary model of the WD was employed, as it is well established to induce obesity and prediabetes conditions in Wistar rats [23,24].

### 3.1. WD Alimentary Model and Heart Function

Our research revealed that in female rats, 18 weeks of WD consumption led to obesity, impaired glucose homeostasis, an altered lipid profile, and elevated fasting leptin levels. WD has also been found to be associated with an increase in both SBP and DBP. These alterations closely mimic key features of MetS and prediabetic conditions in humans [25].

It has been shown that biochemical cardiac remodeling in response to increased glucose oxidation during altered substrate availability reduces metabolic flexibility and contributes to cardiac contractile dysfunction ex vivo [26,27]. However, in our study, echocardiographic assessment of cardiac function in vivo showed no reduction in LV ejection fraction or enlargement of the heart chambers.

It has been demonstrated that obesity can result in increased atrial fibrosis in male mice [28]. Numerous authors have also reported collagen overexpression in the ventricles of rats following a WD and obesity [11,29,30,31]. In contrast, we found that WD does not lead to the development of atrial fibrosis in female rats. Consequently, at the organ and tissue level, there are essentially no structural or functional alterations in the heart, particularly in the atria. These findings are consistent with a recent study by Ponce-Balbuena et al. [32], which showed that diet-induced obesity does not affect the LV ejection fraction or atrial fibrosis in male mice.

### 3.2. Comparison of the WD Model with Other Obesity and Metabolic Disorder Models

Researchers employ a variety of models to induce obesity and metabolic disorders, including HFD, WD, HFD/HSD, the cafeteria diet, and others, with or without added fructose in drinking water. These diets differ significantly in their nutrient compositions and proportions. Additionally, the duration of exposure to these diets varies. The animals used in these experiments also differ in species, strain, and sex. All these factors undoubtedly influence the extent of the resulting metabolic disorders. This aspect requires careful consideration when relating to heart function, mechanics, and calcium dynamics, as the severity of changes can vary significantly based on the extent of the metabolic disorder.

### 3.3. Effect of WD on Contractile Function of Left and Right Atrial Cardiomyocytes

We found that LA cardiomyocytes were significantly more sensitive to the WD than those from the RA. We observed substantial alterations in the morphology, contractile function, and cytosolic [Ca^2+^]_i_ transient amplitude of single LA cardiomyocytes. Specifically, a decrease in both absolute and fractional SL shortening amplitude, along with a reduced maximum SL relengthening velocity, indicated the development of contractile dysfunction and impaired sarcomere relaxation in cardiomyocytes from WD-fed rats. These changes were observed exclusively in the LA, likely due to differences in hemodynamic load between the atria. In contrast, the RA showed no significant changes in the characteristics of sarcomere shortening–relengthening or in ([Ca^2+^]_i_) transient dynamics.

The compensatory hypertrophy of LA cardiomyocytes observed in WD-fed female rats aligns with established clinical data correlating body mass index with LA size in humans [33,34]. This finding is also consistent with experimental models, including mild LA hypertrophy in obese, diabetic db/db mice [35] and LA enlargement in obese sheep [36]. We propose that this hypertrophy likely represents a compensatory response to obesity-induced pressure and volume overload, potentially serving to maintain adequate systemic circulation. However, the concurrent contractile dysfunction and impaired relaxation observed in the LA may ultimately contribute to the development of overt atrial dysfunction.

In contrast to the contractile characteristics of single cardiomyocytes, actin–myosin interaction was disrupted in the LA and RA in the WD group. In the in vitro motility assay, the sliding velocity of NTFs over myosin was reduced for both the RA and LA, indicating a slowdown in the cross-bridge cycle of myosin. Since the isoform composition of myosin remained unchanged, this alteration is likely attributed to post-translational modifications of myosin and thin filament proteins. A significant decrease in cMyBP-C phosphorylation may result in a slowdown in the cross-bridge cycle and contribute to a reduction in cardiomyocyte contractility [37,38].

The slowing of the cross-bridge cycle was more pronounced for the LA and may be the reason for the decrease in the SL shortening amplitude. In the RA, the phosphorylation of RLCs, TnI, and TnT was noticeably reduced in the WD group. This reduction also affects the actin–myosin interaction. The multiple changes in sarcomere protein phosphorylation observed in the RA may reflect early stages of remodeling or could serve as a compensatory mechanism for alterations in contractility.

Changes in cardiomyocyte contractility may also be associated with changes in parameters of cytosolic [Ca^2+^]_i_ transients. We found that WD leads to an increase in calcium amplitude in the LA, which is in good agreement with previous studies [20,32]. An increase in cytosolic [Ca^2+^]_i_ transient amplitude in the LA may be a compensatory mechanism for reduced contractility of cardiomyocytes and may be related to alterations in calcium-handling proteins [39,40,41,42], and it is often associated with atrial fibrillation [43,44]. Previous studies have provided an analysis of calcium signaling in the LA during obesity [20,32]. It was demonstrated that a HFD does not induce changes in the expression of the main the Ca^2+^-handling proteins (the ryanodine receptor; the L-type Ca^2+^ channel, Ca_v_1.2; the Na+/Ca^2+^ exchanger; and SERCA2a) but leads to changes in expression and phosphorylation of phospholamban [32]. In addition, it was found that obesity leads to changes in the T-tubular system in the human and mouse atrial cardiomyocytes [18,20]. We did not detect any changes in the characteristics of Ca^2+^ transients in RA cardiomyocytes. This result suggests that the main Ca^2+^-handling proteins in RA cardiomyocytes in the WD model were unchanged. Verifying this hypothesis should be the goal of future studies.

Differences in the changes in cardiomyocyte contraction in the LA and RA can be attributed to specific variations in gene expression and signaling pathways. In particular, differences in the expression of genes associated with the cytoskeleton, energy metabolism, and regulation of inflammatory processes exist in healthy myocardium and in pathologies between the LA and RA [45,46,47,48,49,50]. There are peculiarities of calcium signaling in the LA and RA [51]. This concept also applies to protein phosphorylation. Consequently, functional remodeling occurs in different ways in the RA and LA. For instance, we previously observed that atrial fibrillation led to more significant structural and functional changes in the LA compared to the RA [52]. Due to its unique functionality, the LA is more sensitive to altered conditions, such as those present in a WD model.

Furthermore, the differences between the LA and RA may be related to the action of sex hormones. For example, in adult rats the expression of mRNA of estrogen receptor-α is significantly higher in the RA than in the LA [53]. It has also been previously demonstrated that the effect of estrogens on the contractile function of cardiomyocytes is chamber-dependent [54,55].

### 3.4. Comparison of the Effect of Obesity on the Contractile Function of the Atrial and Ventricles

In the work of Hegemann et al. [56], ZSF-1 obese rats fed a high-caloric diet for 13 weeks exhibited the HFpEF phenotype. The authors discovered a violation in excitation–contraction coupling and a decrease in cytosolic [Ca^2+^]_i_ transient amplitude in the RV. Hegemann et al. believe that a compensatory increase in myofilament sensitivity is associated with hyperphosphorylation of cMyBP-C.

Desai et al. [57] discovered that in db/db mice with HFpEF, the amplitude of cardiomyocyte contraction, fractional shortening, and the rate of sarcomere contraction increased. However, the characteristics of the Ca^2+^ transient remained unchanged. These alterations in cardiomyocyte contractility were accompanied by hyperphosphorylation of cMyBP-C. The authors did not observe any changes in the level of TnI phosphorylation.

The difference between the findings of these studies and our results can be attributed to the specific characteristics of the obesity models and the study subjects. Additionally, Desai et al. [57] included both male and female rats in their research, whereas Hegemann et al. [56] did not specify the sex of the experimental animals. Despite these variations in experimental models, both studies observed changes in the contractility of cardiomyocytes in the RV and LV, which were associated with an increase in the phosphorylation of cMyBP-C.

In contrast to the findings of the two studies mentioned, our research demonstrates that a decrease in cardiomyocyte contractility in the LA is accompanied by the dephosphorylation of cMyBP-C. This effect may be specific to the chamber. The atria and ventricles contract under different hemodynamic conditions, and there are distinct characteristics in the structure of cardiomyocytes in these chambers, including variations in the isoform composition of sarcomere proteins and calcium signaling proteins. These differences contribute to the unique responses of the atria and ventricles to changes in function during obesity.

## 4. Materials and Methods

### 4.1. Animals and Ethical Approval

All animal experiments were performed in accordance with EU Directive 2010/63/EU of the European Parliament and approved by the ethics committees of the Institute of Immunology and Physiology of RAS (Protocol No. 05/24 from 18 May 2024). Female 8-week-old Wistar rats were obtained from the vivarium of the Institute of Immunology and Physiology. The animals were maintained under standard conditions (12 h light/dark cycle, 22 ± 2 °C, ad libitum access to food and water) throughout the study.

### 4.2. Experimental Design

At the age of 8 weeks, the rats were randomly divided into 2 groups (N = 14 per group): the control group and the WD group. The latter received an experimental diet based on the WD alimentary model [23] with some modifications. The diet included 19% protein, 25% lard, 25% sucrose, and 2% salt [24]. The rats in the control group received a standard rodent diet (Delta Feeds LbK 120 S-19, BioPro, Novosibirsk, Russia).

The experiment was conducted for 18 weeks. Each rat was weighed once a week. At the end of the experiment, all rats were premedicated intramuscularly with 2% Xylazine (1 mL/kg; Alfasan, Woerden, The Netherlands), anesthetized with Zoletil (0.3 mL/kg; Virbac, Carros, France) and rapidly euthanized by exsanguination after heart isolation. The WAT (including gonadal, visceral, retroperitoneal and omental), PAT and liver were also isolated and weighed. The adiposity index was calculated as the ratio of total fat weight to body weight expressed as a percentage.

### 4.3. Oral Glucose Tolerance Test (OGTT)

An OGTT was conducted to evaluate the animals’ ability to handle a glucose challenge after 16 weeks of dietary exposure. The OGTT was performed in unanesthetized rats following 12 h fasting by administration of 20% glucose solution (2 g/kg bw) with a feeding needle, as previously described by Chijiokwu E.A. et al. [58]. Glucose levels were measured with a glucometer (Contur TS, Ascensia Diabetes Care Holdings AG, Basel, Switzerland) using a drop of blood from the tail vein of each rat. The glucose values measured at specific time points (0 (just before), 30, 60 and 120 min after glucose administration) were used to calculate the AUC 0–120 of glucose.

### 4.4. Echocardiography

Echocardiography was performed at 17 weeks of the study under 2% isoflurane (Laboratorios Karizoo, S.A., Barcelona, Spain) anesthesia using a portable ultrasound machine, the GE Vivid iq (GE HealthCare, Chicago, IL, USA), with a 12S-RS 4.5–12 MHz phased array sensor. The following parameters were evaluated: length and width of the RA and RV, LA size at end ventricular systole, end-diastolic and end-systolic sizes of the LV in the parasternal projection, thickness of the interventricular septum (IVS) and LV posterior wall, and Teicholz LV ejection fraction (Th LVEF), as well as the sizes of the pulmonary artery, aortic valve and ascending aorta.

### 4.5. Blood Pressure Measurement

Blood pressure measurements were performed at 17 weeks using a tail-cuff plethysmography system (Systola, Neurobotics, St. Petersburg, Russia). Briefly, conscious rats were conditioned in restraints located on a heating platform-thermostat for rodents (Phlogiston, Neurobotics, Moscow, Russia) controlled at 37 °C for at least 10 min before blood pressure measurement. Rats were habituated for experimental conditions at least 5 consecutive days before data recording.

### 4.6. Biochemical Analysis

Blood collection was performed after 12 h overnight fasting in K3EDTA tubes (VACUETTE^®^ K3E, Greiner bio-one, Frickenhausen, Germany) by cardiac puncture, followed by centrifugation at 1000× *g* for 15 min at 4 °C (Sigma Laboratory Centrifuge 3K30, rotor: 12111–H, Boston, MA, USA). The plasma samples were aliquoted and stored at −80 °C until analysis.

The levels of plasma fasting blood glucose, total cholesterol, triglycerides, and HDL cholesterol were measured by enzymatic colorimetric assays using commercial kits according to the manufacturers’ instructions (Vital Development Corp., St. Petersburg, Russia). The DU-800 spectrophotometer (Beckman Coulter, San Jose, CA, USA) was used to measure absorbance. LDL and VLDL were calculated by means of the Friedewald equation: LDL = Total Cholesterol − HDL − (Triglycerides/2.2), where all values are in mmol/L and (Triglycerides/2.2) is the level of VLDL [59].

### 4.7. Enzyme-Linked Immunosorbent Assay (ELISA)

Fasting plasma levels of C-peptide and leptin were determined using commercially available ELISA kits (Rat C-Peptide ELISA Kit, CSB-E05067r, CUSABIO Inc., Wuhan, China; Invitrogen Rat Leptin ELISA Kit, KRC228, Thermo Fisher Scientific, Inc., Waltham, MA, USA). The assays were conducted in accordance with the manufacturers’ protocols using an automated Lazurite Analyzer (Dynex Technologies, Inc., Chantilly, VA, USA).

### 4.8. Histological Studies

To determine atrial tissue remodeling, hearts were fixed in 10% buffered formaldehyde (pH 7.4) for 48 h, dehydrated in an ethanol series and diaphonized in xylene according to the standard tissue processing protocol using a Leica TP1020 (Leica Microsystems, Wetzlar, Germany). Then samples were embedded in paraffin in a Leica EG1160 embedding station (Leica Microsystems, Germany). The paraffin blocks were subjected to microtomy using a manually operated sliding microtome, the Leica SM2000R (Leica Microsystems, Germany), to obtain 5 µm sections. The frontal longitudinal sections were stained with H&E (Biovitrum, St. Petersburg, Russia) to assess atrial wall thickness. The fractional area of atrial collagen was also determined using picrosirius red staining (Picro Sirius Red Kit, ab150681, Abcam, Cambridge, UK). All measurements were performed at 40× objective magnification using a Leica DM2500 microscope attached to a video camera, the Leica DFC420 (Leica Microsystems, Germany), and connected to a computer equipped with image analysis software (VideoTest Morphology 5.2, St. Petersburg, Russia; Leica Application Suite 4.9, Leica, Germany; ImageJ 1.52).

### 4.9. Cardiomyocyte Isolation

Single atrial cardiomyocytes were isolated using a combined technique of Langendorff perfusion and intra-chamber injections, described in detail elsewhere [52,60]. For both groups, enzymatic digestion of isolated hearts required 12–15 min of whole-heart perfusion on the Langendorff apparatus and 25–30 min of gentle perfusion of the atria via injections. After isolation, cardiomyocytes were sedimented in 5% bovine serum albumin and centrifuged at 200 g for 2 min (rotor: ELMI 6M.05, centrifuge: ELMI CM–6MT, ELMI ltd., Riga, Latvia). Then, extracellular calcium was gradually adjusted, and the cardiomyocyte suspension was stored in HEPES-buffered Tyrode solution (in mM: 140.0 NaCl, 5.4 KCl, 1.0 MgSO_4_, 10.0 HEPES, 11.1 D-glucose, and 1.8 CaCl_2_, pH 7.35, at room temperature with NaOH). Unless otherwise noted, all chemicals and reagents were purchased from Merck (St Louis, MO, USA). Before being used in functional measurements, isolated atrial cardiomyocytes were kept at rest for at least 30 min at room temperature (22 ± 2 °C).

### 4.10. Single Cardiomyocyte Morphometry Measurements

Morphometric parameters (cell length and width) were estimated from images of cardiomyocytes (40× magnification) obtained using MyoCam-S and Ion Wizard 6.6 software (IonOptix Corporation, Milton, MA, USA) and processed offline using FIJI ImageJ 1.52 software (National Institutes of Health, Bethesda, MD, USA).

### 4.11. SL Dynamics Measurements

SL dynamics were monitored during mechanically non-loaded contractions. The average profile of the non-load SL shortening–relengthening was calculated from at least ten steady-state contractions by a fast Fourier transformation-based algorithm using the IonOptix system and Ion Wizard software (IonOptix Corporation, Milton, MA, USA). Measurements were carried out at a stimulation frequency of 1 Hz at 36 ± 1 °C. The following parameters were analyzed: EDSL, absolute sarcomere shortening amplitude (EDSL minus end-systolic SL), absolute and fractional sarcomere shortening amplitude—absolute sarcomere shortening amplitude normalized by EDSL (SL_ampl_), maximum velocities of sarcomere shortening (SL_vshort_) and relengthening (SL_vrel_), TTP, and SL TTR_50_.

### 4.12. [Ca^2+^]_i_ Transient Measurements

Cytosolic [Ca^2+^]_i_ transients were recorded in a narrow region of cardiomyocytes during mechanically non-loaded contractions at 1 Hz and 36 ± 1 °C. CMs were incubated with 1.7 µM Fluo–8AM fluorescent dye (AAT Bioquest, Sunnyvale, CA, USA) and 0.1% Pluronic^®^ F–127 (AAT Bioquest, Sunnyvale, CA, USA) in darkness for 20 min at room temperature, followed by washing with a Tyrode solution. For visualization of cytosolic [Ca^2+^]_i_ transients, a laser scanning microscopic system, the LSM 710, and Zen 2008 software (Carl Zeiss, Jena, Germany) were used. The Fluo–8AM was excited using Ar-laser at 488 nm, and the fluorescence was emitted at 493–575 nm. The changes in the fluorescence signal were calculated as ΔF/F0 values, where F0 is the minimal fluorescence intensity measured between contractions at the diastolic phase of [Ca^2+^]_i_ transients. The following parameters of cytosolic [Ca^2+^]_i_ transients were analyzed using custom-made software, EqapAll 6.0 [61]: [Ca^2+^]_i_ transient amplitude, [Ca^2+^]_i_ TTP and [Ca^2+^]_i_ TTD_50_.

### 4.13. Sarcomeric Protein Preparation, In Vitro Motility Assay, and Phosphorylation Analysis

Sarcomeric proteins were extracted from atrial tissue samples. Atrial tissue specimens were obtained immediately after the chest had been opened and the heart had been washed with PBS. Tissue specimens were frozen in liquid nitrogen and stored at −80 °C until analysis. Cardiac myosin and NTF proteins were extracted according to the standard methods [62].

Sliding-velocity of NTFs over the LA and RA myosin from the control and WD groups at a saturated calcium concentration (pCa 4) were assessed in in vitro motility assay experiments, as described in detail previously [63,64]. The experiments were carried out at 30 °C and repeated 3 times. In each group, 10 image sequences were recorded from different fields. In each field, the movements of 7–12 filaments were tracked for at least 10 frames. The sliding velocities of ~100 actin filaments or NTFs per experiment were measured using the GMimPro2023 software [65].

Isoform composition of myosin heavy chains was determined using 12% SDS-PAGE with SYPRO Ruby staining (Invitrogen, Eugene, OR, USA). Sarcomeric protein phosphorylation was analyzed using 12% SDS-PAGE with Pro-Q Diamond phosphoprotein staining (Invitrogen, Eugene, OR, USA) and SYPRO Ruby staining (Invitrogen, Eugene, OR, USA). The phosphorylation levels of the main sarcomere proteins, cMyBP-C, myosin RLCs, TnT, and TnI, were expressed as a ratio of the Pro-Q Diamond intensity to the SYPRO Ruby intensity. The gel was scanned on the ChemiDoc MP Imaging System (Bio-Rad, Hercules, CA, USA), and band densities were determined with Image Lab 5.2.1 software (Bio-Rad, Hercules, CA, USA).

### 4.14. Statistical Analysis

Statistical analyses and graphics generation were performed using Excel 16 (Microsoft Corp, Redmond, WA, USA) and GraphPrism 10 (GraphPad Software, San Diego, CA, USA). All data were tested for normality using the Shapiro–Wilk test and homogeneity of variances using Bartlett’s test before statistical analysis. The Mann–Witney U-test (non-parametric distribution of data) was used to compare two independent groups (biometric, biochemical, histological, blood pressure and echocardiographic parameters). Nested ANOVA and one-way ANOVA followed by Sidak’s post hoc test (parametric distribution) were used for comparing multiple groups (single cardiomyocytes and contractile protein function). Data were presented as medians (interquartile ranges). The differences were considered statistically significant at *p* < 0.05.

## 5. Conclusions

Our research has clearly demonstrated that 18-week WD promotes moderate obesity, impairment of glucose and lipid homeostasis, and increased blood pressure in female rats. It also has a notable impact on the structural and functional characteristics of left and right atrial cardiomyocytes. Significant changes in contractile function and calcium transient amplitude at this stage affect only LA cardiomyocytes. At the level of contractile proteins, we observed a slowdown in cross-bridge cycling in both atria, regardless of myosin heavy-chain isoform composition, but it was associated with dephosphorylation of the main sarcomere proteins. At the same time, we observed no effects of WD on LV ejection fraction or atrial wall remodeling. Thus, the mid-term WD was responsible for the initial changes in cardiomyocyte contractility. Further studies of the molecular mechanisms in this field considering sex-specific differences may improve understanding of the early stages of cardiovascular pathogenesis in obesity and metabolic disorders. Our pilot study revealed specific changes in the contractile function of single cardiomyocytes in the right and left atria. Further research is needed to elucidate the mechanisms underlying these changes. In particular, questions arise about the effects of obesity on calcium signaling and mitochondrial function in different regions of the heart.

### Limitations

Our study has several methodological limitations. Experiments on isolated LA and RA cardiomyocytes were conducted under mechanically unloaded conditions, even though mechanical load could influence the differences between the LA and the RA and the findings observed. This should be taken into account when interpreting the results. Intracellular calcium ([Ca^2+^]_i_) transient measurement with non-ratiometric dye requires normalization of the signal to resting fluorescence, making it difficult to study diastolic [Ca^2+^]_i_ levels, which is necessary for the comprehension of myocardial stiffness properties.

## Figures and Tables

**Table 1:** The effects of Western diet (WD) on echocardiographic parameters in female rats.

Parameter	Control Group (N = 8)	WD Group (N = 8)	*p*-Value
RA width (mm)	2.0 (2.0; 2.8)	2.0 (2.0; 3.0)	0.645
RA length (mm)	2.5 (2.0; 3.0)	2.5 (2.0; 3.0)	0.878
RV width (mm)	3.0 (2.0; 3.0)	3.0 (2.0; 3.0)	0.798
RV length (mm)	4.0 (4.0; 5.0)	5.0 (5.0; 5.0)	0.195
LA ES size (mm)	2.5 (2.0; 3.0)	3.0 (2.1; 3.0)	0.574
LV ED size (mm)	6.0 (6.0; 6.0)	6.0 (5.3; 6.0)	0.779
LV ES size (mm)	3.0 (3.0; 3.0)	3.0 (2.3; 4.0)	0.779
IVS ES size (mm)	2.0 (2.0; 3.0)	3.0 (2.3; 3.0)	0.152
IVS ED size (mm)	1.0 (1.0; 2.0)	1.5 (1.0; 2.0)	0.867
LVPW ES size (mm)	1.0 (1.0; 2.0)	1.5 (1.0; 2.0)	0.536
LVPW ED size (mm)	2.0 (2.0; 3.0)	2.0 (2.0; 3.0)	0.867
Th LVEF (%)	0.8 (0.8; 0.9)	0.9 (0.7; 0.9)	0.755
Ao valve (mm)	3.0 (2.0; 3.0)	3.0 (2.3; 3.0)	0.613
Ao ascending (mm)	3.0 (2.0; 3.0)	3.0 (3.0; 3.0)	0.336
PA (mm)	2.0 (2.0; 2.3)	2.0 (2.0; 2.8)	0.950

Abbreviations: RA—right atrium, RV—right ventricle, LA—left atrium, ES—end-systolic, LV—left ventricle, ED—end-diastolic, IVS—interventricular septum, LVPW—posterior wall of the left ventricle; Th LVEF—Teicholz LV ejection fraction, Ao—aorta, PA—pulmonary artery, *p*-value—significance level of the Mann–Whitney U-test.

## Data Availability

The original contributions presented in this study are included in the article/Supplementary Materials. Further inquiries can be directed to the corresponding authors.

## Supplementary Materials

The following supporting information can be downloaded at: https://www.mdpi.com/article/10.3390/ijms27031508/s1.

## Abbreviations

The following abbreviations are used in this manuscript:

MetS	Metabolic syndrome
WD	“Western” diet
CVDs	Cardiovascular diseases
HFpEF	Heart failure with preserved ejection fraction
LV	Left ventricular
RV	Right ventricular
SBP	Systolic blood pressure
HFrEF	Heart failure with reduced ejection fraction
HFD	High-fat diet
HSD	High-sugar diet
LA	Left atrium
RA	Right atrium
WAT	White adipose tissue
PAT	Pericardiac adipose tissue
OGTT	Oral glucose tolerance test
AUC	Area under the curve
HDL	High-density lipoprotein
LDL	Low-density lipoproteins
VLDL	Very low-density lipoprotein
DBP	Diastolic blood pressure
H&E	Hematoxylin and eosin
SL	Sarcomere length
EDSL	End-diastolic sarcomere length
SL TTP	Time to peak sarcomere shortening
SL TTR_50_	Time to 50% sarcomere relengthening
[Ca^2+^]_i_ TTP	Time to peak [Ca^2+^]_i_ transient
[Ca^2+^]_i_ TTD_50_	Time to 50% [Ca^2+^]_i_ decay
NTF	Native thin filament
MHCs	Heavy-chain isoforms of myosin
cMyBP-C	Cardiac myosin-binding protein C
RLC	Regulatory light chain of myosin
TnT	Troponin T
TnI	Troponin I
IVS	Interventricular septum
Th LVEF	Teicholz left ventricular ejection fraction
SL ampl	Absolute sarcomere shortening amplitude normalized by EDSL
SL v_short_	Maximum velocities of sarcomere shortening
SL v_rel_	Maximum velocities of sarcomere relengthening
pCa 4	Saturated calcium concentration
