# Supplementary material for: Western Diet-Induced Impairment of Left Atrium Cardiomyocyte Contractility in Female Wistar Rats Is Associated with Slowdown in the Cross-Bridge Cycle and Dephosphorylation of cMyBP-C
Source: Int J Mol Sci. 2026 Feb 3;27(3):1508. doi: 10.3390/ijms27031508 (PMC12898432; doi:10.3390/ijms27031508)
Supplement: Supplementary file 1 [file ijms-27-01508-s001.zip › ijms-4053045-supplementary.pdf]

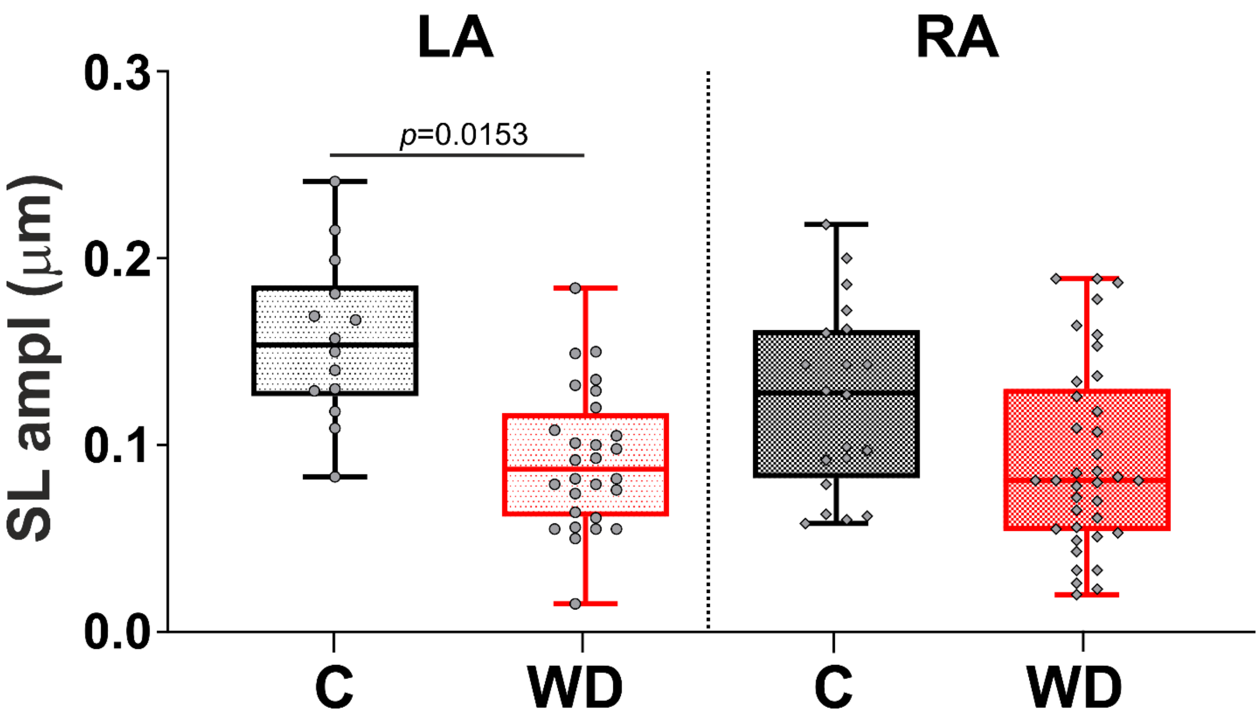

Figure S1. Effects of Western diet (WD) on absolute sarcomere length (SL) shortening amplitude (in μm, SL ampl) in atrial cardiomyocytes. LA – cardiomyocytes of the left atrium; RA – cardiomyocytes of the right atrium. Data are presented in box and whisker plots: the boxes are drawn from Q1 to Q3, bold line shows median, and whiskers provide the 100% range of the values. Each dot represents value of individual cardiomyocyte (N = 6 rats per group). Nested ANOVA followed by Sidak's post hoc test, $p < 0.05$.
